# Application of Self-Attention Generative Adversarial Network for Electromagnetic Imaging in Half-Space

**DOI:** 10.3390/s24072322

**Published:** 2024-04-05

**Authors:** Chien-Ching Chiu, Yang-Han Lee, Po-Hsiang Chen, Ying-Chen Shih, Jiang Hao

**Affiliations:** 1 Department of Electrical and Computer Engineering, Tamkang University, New Taipei City 251301, Taiwan; yhlee@ee.tku.edu.tw (Y.-H.L.); 810440031@gms.tku.edu.tw (P.-H.C.); 409490496@gms.tku.edu.tw (Y.-C.S.); 2 School of Engineering, San Francisco State University, San Francisco, CA 94117-1080, USA; jianghao@sfsu.edu

**Keywords:** inverse scattering problem, self-attention, generative adversarial network, real-time imaging, back-propagation scheme

## Abstract

In this paper, we introduce a novel artificial intelligence technique with an attention mechanism for half-space electromagnetic imaging. A dielectric object in half-space is illuminated by TM (transverse magnetic) waves. Since measurements can only be made in the upper space, the measurement angle will be limited. As a result, we apply a back-propagation scheme (BPS) to generate an initial guessed image from the measured scattered fields for scatterer buried in the lower half-space. This process can effectively reduce the high nonlinearity of the inverse scattering problem. We further input the guessed images into the generative adversarial network (GAN) and the self-attention generative adversarial network (SAGAN), respectively, to compare the reconstruction performance. Numerical results prove that both SAGAN and GAN can reconstruct dielectric objects and the MNIST dataset under same measurement conditions. Our analysis also reveals that SAGAN is able to reconstruct electromagnetic images more accurately and efficiently than GAN.

## 1. Introduction

Electromagnetic imaging is a sensor technique used in various fields, including medical imaging, remote sensing, and security applications. However, electromagnetic imaging is an emerging technology that has drawn a lot of attention recently. It can be used in many arenas, such as surface exploration, medical imaging, and so on. Generally, two major techniques are used to solve the electromagnetic imaging problems: (1) traditional algorithms and (2) artificial intelligence. In the study of traditional algorithms, they can further be classified by the two main types of algorithms [1,2,3]: (1) iterative algorithms, such as the distorted Born iterative method and the distorted Born approximation, etc., and (2) non-iterative algorithms, such as the Born approximation (BA) and the Rytov approximation (RA), etc. While in the artificial intelligence method, it can be used as an approximation method for the initial input image. In the AI mechanism, the data input to the neural network can be categorized as (1) scattered field input [4,5,6] and (2) initial shape (or dielectric) guess input [7,8,9,10,11,12,13,14]. In 2019, Yao introduced a two-stage neural network architecture to deal with the inverse scattering problem. The initial dielectric coefficient distribution was first estimated by inputting the measured scattered field into a complex value deep convolutional neural network. In the second stage, the initial dielectric coefficient distribution obtained from the complex value deep convolutional neural network in the first stage was further input into a deep residual convolutional neural network to reconstruct an accurate electromagnetic image [4]. In 2020, Yao proposed a deep convolutional neural network to tackle the electromagnetic inverse scattering problem. Numerical results showed that this method could effectively reconstruct high-contrast scatterers [5]. Well-reconstructed results have been obtained by this method. In 2023, Zhang input a single-frequency scattered field into the deep residual convolutional neural network to expand to multifrequency. This scattered field was next input to a deep convolutional encoder–decoder for electromagnetic imaging [6]. Numerical results showed that the reconstruction was good.

In 2020, Xu compared three different input training schemes for Convolutional Neural Networks (CNN): the direct inverse scheme, phaseless data-dominant-induced currents, and phaseless data contrast source inversion. Numerical results showed that phaseless data contrast source inversion had better accuracy and generalization ability [7]. In 2021, Guo proposed a novel GAN to improve the resolution of the preliminary images. Compared with the traditional optimized mechanism, this method exposed better computation performance and resolution [8]. In 2022, Liu proposed two physically oriented loss functions to improve the noise immunity as well as the resolution of the reconstructed images for deep learning [9]. Also in 2022, Liu proposed a generative adversarial network for point cloud upsampling. Results showed that the visual quality of the upsampled point clouds produced by this method is superior to current state-of-the-art methods [10]. In 2023, Wang proposed an early fusion deep learning framework for solving the electromagnetic inverse scattering problem. The accuracy of the reconstructed image was improved by fusing the input data and the noise immunity was enhanced. Numerical results demonstrate the effectiveness of the proposed method [11]. To conclude, the first method that inputs the scattered field is able to reconstruct high-contrast scatterers more rapidly but with low resolution. Nevertheless, the second method that inputs the initial dielectric constant guess image takes plenty of time to reconstruct a high-resolution image but not for high-contrast scatterers.

In recent years, attention mechanisms have found widespread application in artificial intelligence technologies, particularly in the field of image processing [15,16,17,18]. Attention mechanism is a technique that integrates human behaviors into deep learning, allowing computers to discern the significance of data through the perceptual understanding. In 2022, Li proposed a GAN with local and global attention mechanisms to enhance the resolution of remote sensing images. Numerical results validated that the integration of a global attention mechanism in the generative network captured correlations between channel and spatial dimensions and optimized the generated images. Moreover, the network discriminative capabilities had also been improved simultaneously [15]. In 2022, Xu proposed an attention GAN to remove bright spots in a single gray-scale image and compared it with other generative adversarial network methods to confirm the effectiveness of this method [16]. In 2023, Xu introduced a Fourier Bases Expansion of Contraction Integral Equation algorithm (FBE-CIE-I) combined with GAN architecture and attention mechanism to solve electromagnetic inverse scattering problems. Numerical results demonstrated that the initial image obtained through FBE-CIE-I could effectively capture low-frequency components, aiding GAN to regenerate higher-frequency components. In other words, incorporating attention mechanisms at the end of the generative network could seize the physical distance information between pixels efficiently and, hence, increase the resolution of the reconstructed images [17]. In 2023, Wang proposed a U-shaped network with mixed attention for reconstructing remote sensing images. Numerical results indicated that the proposed method had effectively utilized an attention mechanism in convolutional layers to extract global features [18].

In recent years, some relevant research has been published in half-space object detection. In 2012, Pastorino introduced the Newton algorithm for reconstructing buried objects by employing numerical simulations [19]. In 2019, Chiu employed Self-Adaptive Dynamic Differential Evolution (SADDE) to regenerate buried dielectric objects under non-flat rough surfaces [20]. Continuing this trend, Huang presented a Full-Wave Inversion (FWI) method for buried anisotropic objects in 2021. Notably, this cascading inversion scheme led to significant cost savings in computational expenses [21]. Expanding on these advancements, Liang proposed the variational Born iteration technique in 2022 to reconstruct targets within layered composite structures. Leveraging multiple orbital angular momentum modes, this method notably enhanced the accuracy and quality of reconstruction [22]. Despite these significant strides, it remains a common challenge across these methodologies that they require substantial time for computing recurring complex Green’s functions.

Ground-Penetrating Radar (GPR) is commonly employed in both the time and frequency domains [23,24,25]. In 2018, Ozkaya introduced a groundbreaking algorithm applying a multi-level deep learning approach for detecting buried objects in GPR B-scans. This method implemented a layer-by-layer training strategy to construct deep dictionaries capturing the features of buried objects. Subsequently, various classifiers used these dictionaries to identify and classify the detected objects accurately and significantly [23]. In 2022, Barkataki presented a CNN model to predict the size of buried objects from GPR B-scans. Promising results had been attained [24]. Wang presented an innovative inversion method using a Deep Neural Network inverse approach to estimate the relative permittivity of a target. The proposed method’s reliability was evaluated via a GPR simulation dataset as well as a dataset of underground rainwater pipes. Results indicated that DNN-based inversion method was a reliable and accurate approach for determining the relative dielectric constant, marking a prominent development for real-life underground pipe inspection [25]. It is noteworthy that previous studies were predominantly focused on reconstructing the position or size of the objects, neglecting the dielectric permittivity aspect. GPR typically employs time–domain pulses of electromagnetic waves at various frequencies to irradiate buried objects and soil layers. In contrast, our approach utilizes a time harmonic field that transmits electromagnetic waves at a single frequency for reconstruction, which, generally, may encounter additional challenges in frequency domain.

**Figure 1:**
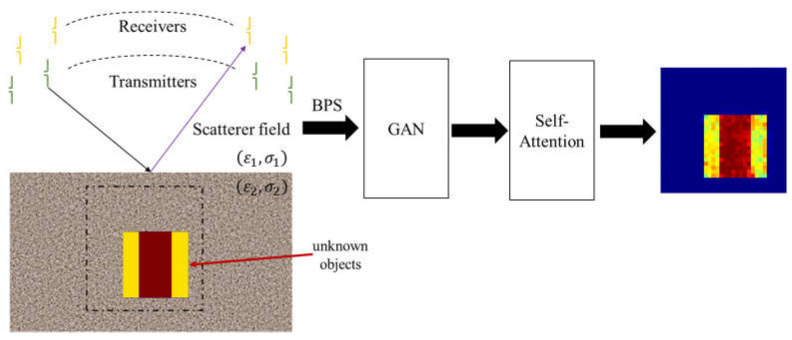
Sensing and reconstruction architecture.

Our system architecture is shown in Figure 1. The transmitting antennas are half-wave dipoles and receiving antennas are also half-wave dipoles. The figure shows that we place the transmitters to illuminate the unknown objects and receivers to record the scattered field in a simulated environment. Next, we use the measured scattering field information to estimate the initial image through BPS. Finally, this estimated image is input to GAN with the self-attention mechanism block to reconstruct the ground truth image.

The contributions of this work include the following:

1. To the best of our knowledge, there is no half-space electromagnetic imaging publication so far for SAGAN. In this article, we propose SAGAN to solve highly nonlinear inverse scattering problems. Since measurements can only be made in the upper space, the measurement angle will be limited. Numerical results show that our proposed method is capable of producing fast and accurate imaging, specifically for highly nonlinear scatterers.
2. We have successfully implemented GAN and SAGAN to reconstruct electromagnetic images buried in half-space and compared their performance. In the SAGAN model, we design a hybrid loss function in the generator network to improve the quality of the reconstructed image. Furthermore, the self-attention module is used for regularizing the physical equations and mimicking the multiple scattering effect in modeling.
3. In the numerical results, we analyze the reconstruction effect of the self-attention mechanism in electromagnetic imaging. To verify the effectiveness of our proposed method, we use the trained model to reconstruct the case of high-permittivity distribution. Results showed that our proposed method is still highly reliable in the half-space environment.
4. By training the network model in advance with appropriate parameter configuration, we can obtain the results rapidly by inputting new data into the model. In other words, we use the trained SAGAN to recover high-resolution electromagnetic imaging in half-space effectively.

We introduce the theory and formulas in Section 2. GAN and SAGAN architecture are described in Section 3. Section 4 analyzes the numerical results. Conclusions are given in Section 5.

## 2. Theory and Formulas

### 2.1. Direct Problems

**Figure 2:**
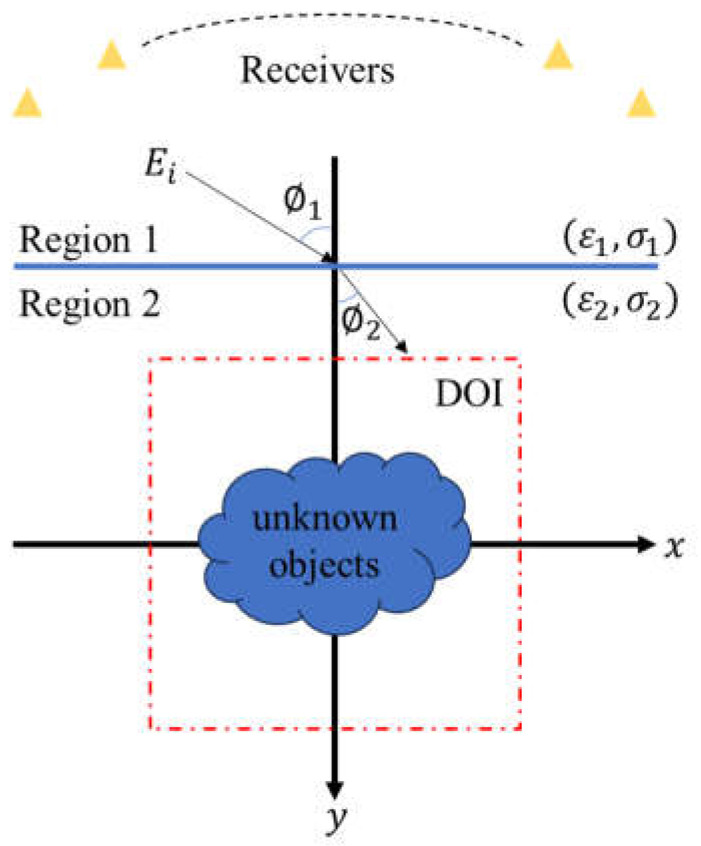
Schematic diagram of a two-dimensional object buried in a half-space.

Considering a dielectric object located in a lossy half-space, as illustrated in Figure 2, $\left(\varepsilon_{1},\sigma_{1}\right)$ and $\left(\varepsilon_{2},\sigma_{2}\right)$ denote the permittivity and conductivity in Region 1 and Region 2, respectively. Let $\mu_{0}$ be the permeability of free space in each region. In other words, non-magnetic substances are solely regarded here. The scatterer is a dielectric object extending in the *z*-axis infinitely. The time-varying relation of the incident wave is set to $e^{j\omega t}$ and its incident angle is $\emptyset_{1}$.

To streamline the analysis, we assume the TM wave is polarized parallel to the *z*-axis. The electric field distributed in the absence of scatterers is denoted as $E_{i}$ and can be expressed mathematically as follows:

(1) $${\vec{E}}_{i}\left(\vec{r}\right)=E_{i}\left(x,y\right)\hat{z}=\left\{\begin{array}{l} E_{1}\left(x,y\right)=e^{-jk_{1}[x{\mathrm{sin\varphi}}_{1}+(y+a){\mathrm{cos\varphi}}_{1}]}+R_{1}e^{-jk_{1}[x{\mathrm{sin\varphi}}_{1}-(y+a){\mathrm{cos\varphi}}_{1}]},y\leq -a\\ E_{2}\left(x,y\right)=Te^{-jk_{2}[x{\mathrm{sin\varphi}}_{2}+(y+a){\mathrm{cos\varphi}}_{2}]}\;\;\;\;\;\;\;\;\;\;\;\;\;\;\;\;\;\;\;\;\;\;,y>-a \end{array}\right.$$

where

(2) $$R_{1}=\frac{1-n}{1+n},\;\;\;T=\frac{2}{1+n},\;\;\;n=\frac{{cos\varphi }_{2}}{{cos\varphi }_{1}}\sqrt{\frac{\varepsilon_{2}-j\sigma_{2}/\omega }{\varepsilon_{1}-j\sigma_{1}/\omega }}$$

(3) $$k_{1}sin\varphi_{1}=k_{2}sin\varphi_{2}$$

(4) $$k_{i}^{2}=\omega^{2}\varepsilon_{i}\mu_{0}-j\omega \mu_{0}\sigma_{i}\;\;,\;\;i=1,2\;\;Im(k_{i})\leq 0$$

If Regions 1 and 2 consist of lossless media, then $\varphi_{1}$ and $\varphi_{2}\;$indicate the incident and refracted angles, respectively. Conversely, if Regions 1 and 2 involve lossy media, the angles $\varphi_{1}$ and $\varphi_{2}$ become more intricate.

Based on the concept of induced current and Maxwell’s equations, the following equations are derived:

(5) $$\nabla \times \overset{⃑}{E}=-j\omega \mu_{0}\overset{⃑}{H}$$

(6) $$\nabla \times \overset{⃑}{H}=j\omega \varepsilon_{2}\overset{⃑}{E}+{\overset{⃑}{j}}_{eq}$$

where ${\overset{⃑}{j}}_{eq}=j\omega \varepsilon_{0}\left[\varepsilon_{r}\left(x,y\right)-\varepsilon_{2}\right]E\hat{z}$ is the equivalent current density of the dielectric object.

The total electric field inside the object ${\vec{E}}_{t}\left(x,y\right)=E_{t}\left(x,y\right)\hat{z}=\left[E_{i}\left(x,y\right)+E_{s}\left(x,y\right)\right]\hat{z}$ can be expressed by the two-dimensional Green’s function as

(7) $$E_{i}\left(\bar{r}\right)=\int_{s}G\left(r,r^{\prime }\right)k_{2}^{2}\left[\varepsilon_{r}\left(r^{\prime }\right)-\varepsilon_{2}\right]E_{t}\left(r^{\prime }\right)ds^{\prime },\;\;y>-a$$

The scattered field can be written as

(8) $$E_{s}\left(\bar{r}\right)=-\int_{s}G\left(r,r^{\prime }\right)k_{2}^{2}\left[\varepsilon_{r}\left(r^{\prime }\right)-\varepsilon_{2}\right]E_{t}\left(r^{\prime }\right)ds^{\prime }$$

To address this half-space problem, the Green’s function, denoted as $G(x,\;y;\;x^{\prime },\;y^{\prime })$, needs to be initially solved. This involves utilizing a line current source at $\left(x^{\prime },\;y^{\prime }\right)$ and determining the scattered field at (x, y). Employing the Fourier transform technique, the half-space Green’s function $G(x,\;y;\;x^{\prime },\;y^{\prime })$ can be expressed as follows:

(9) $$G\left(x,y{;x}^{\prime },y^{\prime }\right)=\left\{\begin{array}{l} G_{1}\left(x,y{;x}^{\prime },y^{\prime }\right)\;\;\;\;\;\;\;\;\;\;\;\;\;\;\;\;\;\;\;\;\;\;\;,\;y\leq -a\\ G_{2}\left(x,y{;x}^{\prime },y^{\prime }\right)=G_{f}\left(x,y{;x}^{\prime },y^{\prime }\right)+G_{s}\left(x,y{;x}^{\prime },y^{\prime }\right)\;\;\;\;,y>-a \end{array}\right.$$

(10) $$G_{1}\left(x,y{;x}^{\prime },y^{\prime }\right)=\frac{1}{2\pi }\int_{-\infty }^{\infty }\frac{j}{\gamma_{1}+\gamma_{2}}e^{j\gamma_{1}\left(y+a\right)}e^{-j\gamma_{2}\left(y\prime +a\right)}e^{-j\alpha \left(x-x^{\prime }\right)}d\alpha$$

(11a) $$G_{f}\left(x,y{;x}^{\prime },y^{\prime }\right)=\frac{j}{4}H_{0}^{\left(2\right)}\left[k_{2}\sqrt{(x-x\prime {)}^{2}+(y-y\prime {)}^{2}}\right]$$

(11b) $$G_{s}\left(x,y{;x}^{\prime },y^{\prime }\right)=\frac{1}{2\pi }\int_{-\infty }^{\infty }\frac{j}{2\gamma_{2}}\left(\frac{\gamma_{2}-\gamma_{1}}{\gamma_{2}+\gamma_{1}}\right)e^{-j\gamma_{2}\left(y+2a+y^{\prime }\right)}e^{-j\alpha \left(x-x^{\prime }\right)}d\alpha$$

(12) γi2=ki2−α2,  i=1,2 ,  Im⁡(γi)≤0 

Here, $k_{i}$ denotes the wave number of the *i*-th region, and $G(x,\;y;\;x^{\prime },\;y^{\prime })\;$represents the half-space Green’s function, acquired through the Fourier transform in (11a), where $H_{0}^{2}$ stands for the second-order zero Hankel function. In the numerical solution of Equations (7) and (8), it is crucial to calculate the Green’s function in Equation (9). However, in situations where the points (x, y) and $\left(x^{\prime },\;y^{\prime }\right)$ closely approach the interface between the two regions at y=−a, the convergence in the integration process becomes sluggish. Consequently, this results in a substantial computational burden for evaluating the half-space Green’s function in such cases.

### 2.2. Back Propagation Scheme

In this section, we perform a non-iterative inversion method before training the neural network to reconstruct the permittivity distribution of the buried object. For the sake of efficiency for GAN and SAGAN, BPS is first applied to compute the initial permittivity distribution via the measured scattered field information. The induced current $I^{b}$ is assumed to be proportional to the back-propagation field, where χ is a constant and H denotes the conjugate transpose.

(13) $$I^{b}=\chi \cdot {\left[G_{1}\right]}^{H}\left(E_{s}\right)\;$$

The loss function $F^{b}$ is then defined as

(14) $$F^{b}\left(\chi \right)={\left\|\left(E_{s}\right)-\left[G_{1}\right]\cdot \chi \cdot {\left[G_{1}\right]}^{H}\left(E_{s}\right)\right\|}^{2}\;$$

To ascertain the minimum value of F(χ), we set the derivative of F(χ) to zero. The analytical solution for χ can be expressed as follows:

(15) $$\chi_{m}=\frac{{\left(E_{s}\right)}^{T}\cdot {\left(\left[G_{1}\right]\left({\left[G_{1}\right]}^{H}\cdot \left(E_{s}\right)\right)\right)}^{*}}{{\left\|\left[G_{1}\right]\left({\left[G_{1}\right]}^{H}\cdot \left(E_{s}\right)\right)\right\|}^{2}}$$

where T and * represent the transpose and complex conjugate, respectively.

We can derive the induced current using Equation (13), upon which $\chi_{m}$ is determined. Subsequently, the total back-propagation field $E_{z}^{b}$ can be defined as

(16) $$\left(E_{t}^{b}\right)=\left(E_{i}\right)+\left[G_{1}\right]\left(I^{b}\right)$$

Based on the definition of the induced current, the dielectric coefficient (τ) and the induced current $I^{b}$ can be written as follows:

(17) $$I_{p}^{b}=diag\left(\left[\tau_{z}^{b}\right]\right)\left(E_{t}^{b}\right)$$

where p represents the incidence at each different angle.

By employing the least-squares problem technique to combine all instances of Equation (17), the analytical solution for each element can be derived as follows:

(18) $$\left[\tau_{z}^{b}\right]\left(n\right)=\frac{\sum_{\rho =1}^{Mi}I_{p}^{b}\left(n\right)\cdot {\left[\left(E_{t}^{b}\right)\left(n\right)\right]}^{*}}{\sum_{\rho =1}^{Mi}{\left\|\left(E_{t}^{b}\right)\left(n\right)\right\|}^{2}}$$

where Mi is the number of incidences.

## 3. Neural Network

This powerful deep learning model trains two neural networks, the generator and discriminator, in a competitive manner. It has achieved great success in various deployments, including image generation, style migration, image-to-image translation, etc. Training GANs is challenging due to the issues of instability and gradient vanishing, etc. Many variants of GAN models with enhanced stability, scalability, and a range of applications have been proposed recently. These advances have strengthened the position of GAN as an important tool in the field of generative modelling and artificial intelligence.

**Figure 3:**
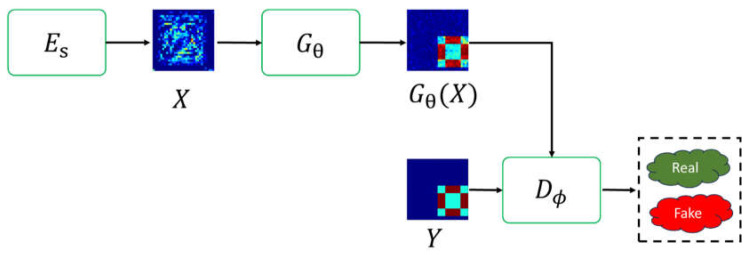
GAN architecture.

In this paper, the GAN depicted in Figure 3 is referred to as $G_{\theta }$ and $D_{\emptyset }$ for the generator and discriminative network, with θ and ∅ representing the unknown parameters of the generator and discriminative network.

**Figure 4:**
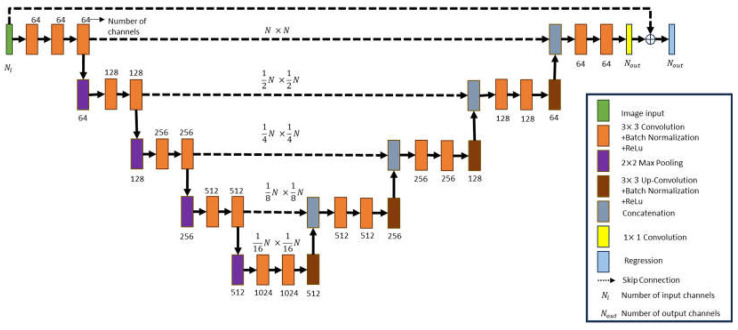
The schematic diagram for the GAN generator.

As shown in Figure 4, a contracted network, an expanded network, the repeatedly 3 × 3 convolution layers, Batch Normalization layers, as well as the ReLU layers are united to form the U-Net structure GAN generator. The shrink network pooling layer utilizes a 2 × 2 max-pooling layer, while the expanded network pooling layer employs a 3 × 3 transposed convolution layer. Lastly, a 1 × 1 convolution is employed in the fully connected layer. $N_{i}$, the number of incidences, is equal to $N_{out}$, the number of output channels. The regression layer takes the average output from the fully connected layer to compute the error value of the dielectric coefficient distribution.

**Figure 5:**
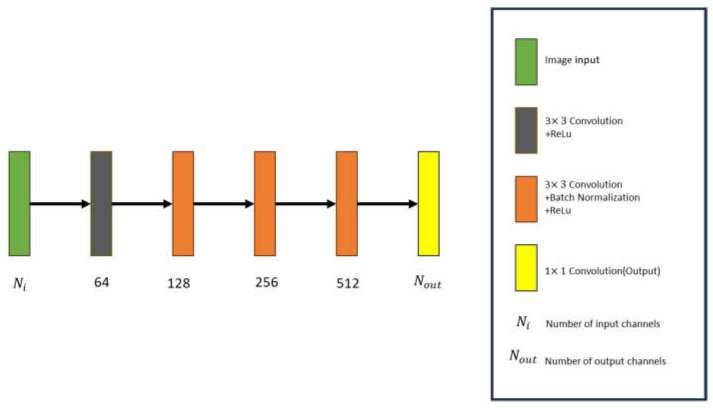
The schematic diagram for the GAN discriminator.

The discriminative network produces a discriminative matrix as its output. The generative network and the discriminative network undergo alternating and mutually exclusive training. The discriminator’s architecture is composed of iteratively adding convolution layers, Batch Normalization layers, and ReLU layers, as illustrated in Figure 5. The input data for the discriminator is the image generated by the generative network. Essentially, the discriminator evaluates the generated image and assigns a score, determining whether the generative network should update its training weights. This iterative process continues until a satisfactory balance is achieved.

The loss function of the generative network $L_{GAN}^{G}$ can be defined as

(19) $$L_{GAN}^{G}\left(\theta |\emptyset \right)=L_{RMSE}\left(\theta \right)+\gamma L_{A}\left(\theta |\emptyset \right)$$

Here, $L_{RMSE}\;(\theta )$ represents the error between the reconstructed image and the reference image. We define the Root-Mean-Square Error (RMSE) formula as follows:

(20) $$L_{RMSE}=\frac{1}{M}\sum_{i=1}^{M}\frac{{\left\|I-I^{\alpha }\right\|}_{F}}{{\left\|I\right\|}_{F}}$$

where I and $I^{\alpha }$ represent the true and reconstructed shapes, respectively, M is the number of tests conducted, and F depicts the Frobenius norm, with γ being the weight parameter used to balance these two losses.

(21) $$L_{A}\left(\theta |\emptyset \right)=\frac{1}{N}\sum_{i=1}^{N}{\left\|D_{\emptyset }\left(G_{\theta }\left(X_{i}\right)\right)-1\right\|}_{1}$$

$L_{A}$ serves as the scoring mechanism of the discriminative network to assess the authenticity of the overall reconstructed image. *N* represents the size of the data input into the batch.

The loss function of the discriminative network can be expressed as

(22) $$L_{GAN}^{D}\left(\theta |\emptyset \right)=\frac{1}{2N}\sum_{i=1}^{N}\left(\left\|D_{\emptyset }\left(Y_{i}\right)-1\right\|\begin{array}{c} 2\\ 2 \end{array}+\left\|D_{\emptyset }\left(G_{\theta }\left(X_{i}\right)\right)\right\|\begin{array}{c} 2\\ 2 \end{array}\right)$$

Here, ∅ represents the unknown parametric data, and θ is the weight parameter. $Y_{i}$ and $X_{i}$ denote the true and trained data, respectively. The optimization process alternately focuses on $D_{\emptyset }$ and $G_{\theta }$ in an adversarial manner until a Nash equilibrium is reached. In other words, the process will cease when the data generated by the generator $G_{\theta }\left(X\right)$ closely resemble the real image and can no longer be distinguished from the authentic data by the discriminator $D_{\emptyset }$.

The SAGAN functions as a generative model. Self-attention is a neural network mechanism utilized to assess the significance of various segments within the input sequence during the processing of each element. Briefly speaking, it serves as a potent tool for capturing relationships within sequences and refines the performance of the neural network models across various domains. The key concepts of self-attention are summarized as follows: (1) Attention scores: In self-attention, attention scores are computed for each element in the input sequence concerning other elements. These scores ascertain the importance or relevance of each element during the processing of a specific element. (2) Weights and context vectors: The attention scores undergo transformation into weights via a SoftMax function, creating a probability distribution. Subsequently, these weights are used to compute a weighted sum of the input sequence, resulting in a context vector. The context vector accentuates elements more pertinent to the current position. (3) Parallelization and efficiency: Self-attention enables the parallelization of computation, allowing each element to independently attend to all other elements. In short, this parallelization allows high-performance computing, especially for lengthy sequences, in contrast to the traditional sequential approaches. (4) Capture of Long-Range Dependencies: A notable advantage of self-attention lies in its capability to capture long-range dependencies within sequences. Traditional recurrent neural networks may encounter challenges with dependencies distributing far apart in the sequence, whereas self-attention can comprehensively consider all positions. The nonlinearity inherent in inverse scattering is widely recognized to be influenced by the multiple scattering effects of the degree of interest. In this context, the induced current at each pixel gives rise to a scattered field at another pixel, and this relationship hinges on the distance separating the two pixels. Consequently, the application of self-attention emerges as a valuable approach to capture the physical distance information between two pixels, enhancing the model’s ability to grasp the intricacies of the multiple scattering process.

**Figure 6:**
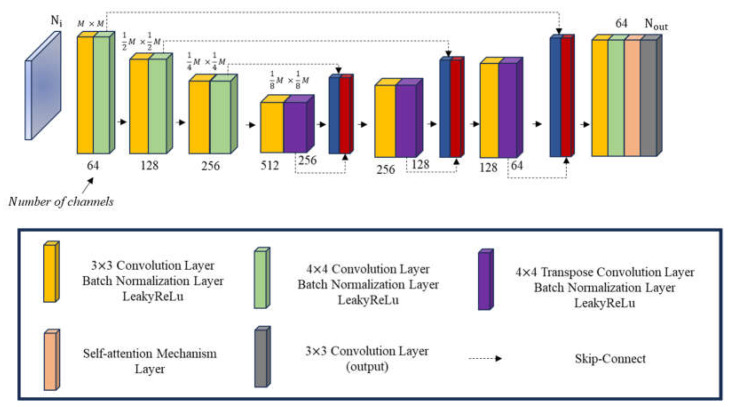
The schematic diagram for the SAGAN generator.

**Figure 7:**
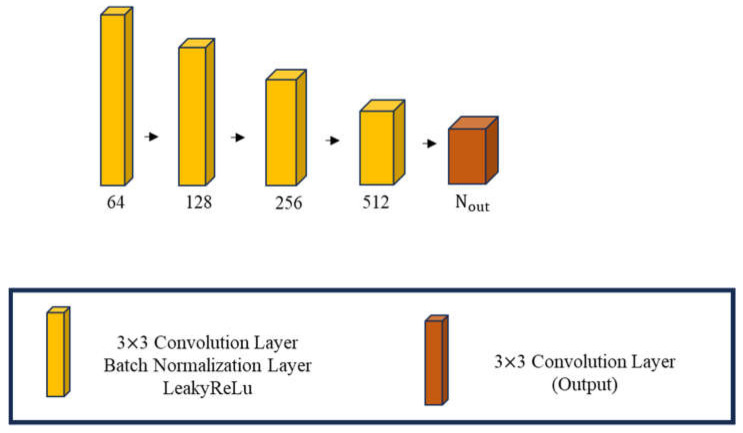
The schematic diagram for the SAGAN discriminator.

In this research, we have merged GAN with attention mechanisms into SAGAN. This amalgamation enhances the network’s capacity to capture distant dependencies, leading to the generation of more realistic and coherent data. The SAGAN architecture with the generator and discriminator, respectively, is shown in Figure 6 and Figure 7.

**Figure 8:**
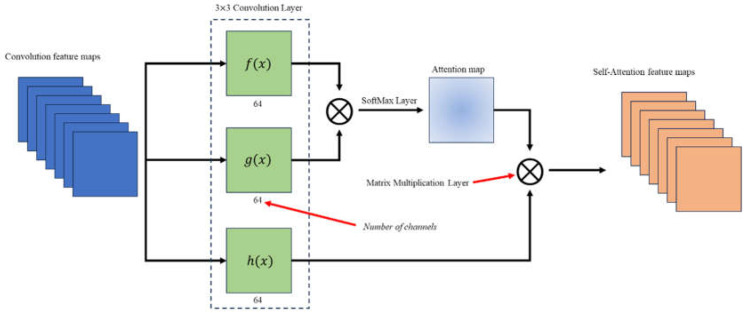
Self-attention architecture.

As shown in Figure 6, the SAGAN generator comprises a contracting network on the left half and an expanding network on the right half. The contracting network incorporates continuously added 3 × 3 convolution layers, Batch Normalization layers, and LeakyReLU layers. During the contraction phase, 4 × 4 convolution layers, Batch Normalization layers, and LeakyReLU layers are employed. While in the expansion phase, 3 × 3 transposed convolution layers with Batch Normalization layers and LeakyReLU layers are utilized for pooling. Subsequently, 3 × 3 convolution layers, Batch Normalization layers, and LeakyReLU layers are introduced to extract features. A self-attention layer is introduced just before the final output to enable the neural network to capture long-range dependencies, resulting in more realistic and coherent data recovery, as illustrated in Figure 8. The ultimate 1 × 1 convolution serves as the generator’s output to be fed into the discriminator for distinguishing between True and False. The input channel number ($N_{i}$) should align with the output channel number ($N_{out}$).

The discriminative network generates a discriminative matrix as its output, in which its training alternates with the generative network in an exclusive manner. The discriminator’s structure is formed by iteratively incorporating convolution layers, Batch Normalization layers, and LeakyReLU layers, as depicted in Figure 7. The input data of the discriminator consist of images created by the generative network. In essence, the discriminator assesses each generated image, assigning a score that dictates whether the generative network should adjust its training weights. This cyclic process persists until a satisfactory equilibrium is attained.

For the objective of generating images with comparable features, it is essential to note that they collect distinct types of features. $L_{RMSE}$ strives to produce images with features akin to the target image, obtained through convolution kernels, and incorporating details like edges and gradients. On the other hand, $L_{SSIM}$ gauges the perceptual distance between two images by considering factors such as luminance, contrast, and structural information. In light of this, the integration of SAGAN and SSIM is undertaken to reinforce the similarity between the generated image and the target image by incorporating the strengths of both approaches. The loss function of the generative network by SAGAN $L_{SAGAN}^{G}$ can be defined as follows:

(23) $$L_{SAGAN}^{G}\left(\theta |\emptyset \right)={L_{RMSE}+\lambda_{1}\cdot L}_{A}+\lambda_{2}\cdot L_{ssim}$$

(24) $$L_{ssim}=1-SSIM(\tilde{y},y)$$

(25) $$SSIM\left(\tilde{y},y\right)=\frac{\left(2\mu_{\tilde{y}}\mu_{y}+C_{1}\right)\left(2\sigma_{\tilde{y}y}+C_{2}\right)}{\left(\mu_{\tilde{y}}^{2}+\mu_{y}^{2}+C_{1}\right)\left(\sigma_{\tilde{y}}^{2}+\sigma_{y}^{2}+C_{2}\right)}$$

Where $\tilde{y}$ and *y* denote, respectively, the reconstructed and true relative permittivity profiles; $\mu_{y}$ and $\sigma_{\tilde{y}}^{2}$ are the mean and variance of *y*, respectively; and $\sigma_{\tilde{y}y}$ denotes the covariance for $\tilde{y}$ and *y*. To prevent a zero denominator, two small constraints, $C_{1}={\left(K_{1}D\right)}^{2}$ and $C_{2}={\left(K_{2}D\right)}^{2}$, with $K_{1}=0.01$ and $K_{2}=0.03$ as the two hyperparameters with D, the dynamic range of pixel values for the target object *y*, have been considered.

## 4. Numerical Results

This section details the design of a simulation environment to analyze inverse scattering problems for dielectric objects buried in half-space, as depicted in Figure 2. The incident wave frequency is set at 3 GHz and the scatterers are illuminated by TM waves. To emulate real-world conditions, 10% and 20% Gaussian noise are added in the simulation environment, respectively. A configuration comprising 32 receivers spanning from θ= $195^{o}$ to $350^{o}$ with a radius of distance set at 3 m and 32 transmitters ranging from $\emptyset_{1}=$ ${-80}^{o}$ to $80^{o}$ at $5^{o}$ intervals are employed. In this simulation environment, 10 scatterers with different permittivity distributions are randomly placed at 50 different positions in a 32 × 32-pixel area. By measuring the scattered field, an initial image is guessed via BPS. The dataset comprises approximately 500 images, with 90% for training, 5% for validating, and 5% for testing.

During the training process, each iteration is conducted independently, and GPU parallelization is applied to enhance the computational efficiency. The initial learning rate parameter for Adaptive Moment Estimation (ADAM) is set to 0.0002. The batch size and maximum number of epochs are configured as 16 and 200, respectively. To boost training effectiveness using GAN and SAGAN, data shuffling is executed after each epoch. Note that the same training parameters and noise level are applied to compare the performance of GAN and SAGAN in our simulations. 

Equations (20) and (25) are used to evaluate the reconstruction results trained by GAN and SAGAN.

### 4.1. GAN and SAGAN Performance Comparison for Reconstruction Permittivity Between 3 and 3.5 with 20% Noise Level

**Figure 9:**
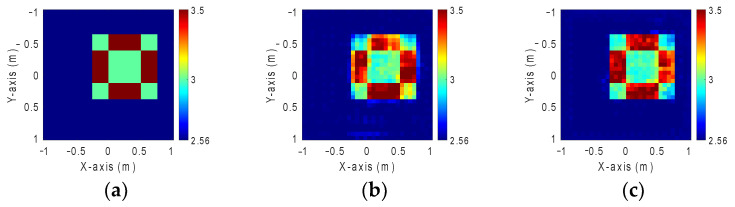
Permittivity from 3 to 3.5. (**a**) Ground truth. (**b**) Reconstructed image by GAN with 20% noise. (**c**) Reconstructed image by SAGAN with 20% noise.

In this context, we define the dielectric constant distribution to range between 3 and 3.5. Our simulated environment consists of 32 transmitters and receivers. To emulate real-world conditions, we add 20% Gaussian noise into the measured scattered field. We postulate that scatterers exhibit 10 distinct dielectric constant distributions and can be randomly positioned at any of the 50 locations in the measurement compound. Consequently, the dataset for each scenario comprises a total of 500 images. We partition the dataset into three subsets with 90% for training, 5% for validating, and 5% for testing to facilitate the process. BPS methods are implemented to estimate the initial dielectric constant distribution. This estimated distribution is then input into both GAN and SAGAN models for comparative analysis. Figure 9a shows the ground truth image. Figure 9b,c show the reconstructed image by GAN and SAGAN with a 20% noise level. The RMSE and SSIM are listed in Table 1. It is inspiring to report that SAGAN demonstrates greater accuracy and clarity in reconstructing both the shape and dielectric constant of the objects compared to GAN.

### 4.2. GAN and SAGAN Performance Comparison for Reconstruction Permittivity Between 3.5 and 4 with 10% Noise Level

**Figure 10:**
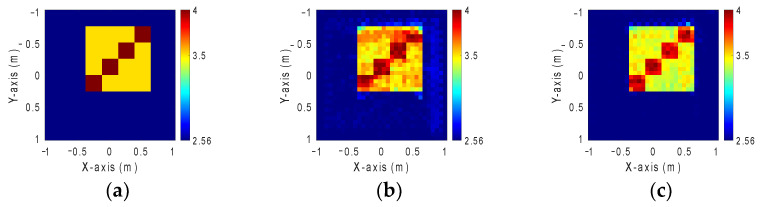
Permittivity from 3.5 to 4. (**a**) Ground truth. (**b**) Reconstructed image by GAN with 10% noise. (**c**) Reconstructed image by SAGAN with 10% noise.

In this study, we define the dielectric constant distribution within the range from 3.5 to 4. Like before, our simulation environment comprises 32 transmitters and receivers. This time, we introduce 10% Gaussian noise into the measured scattered field to replicate a realistic environment. Again, 10 different dielectric constant distributions are presumed and are randomly placed at any 50 locations within the measurement area to come out to 500 images in total. For simplicity, we split the dataset into 90% for training, 5% for validating, and 5% for testing. To estimate the initial dielectric constant distribution, we employ the BPS method. This estimated distribution is next input into GAN and SAGAN models for comparative analysis. Figure 10a displays the ground truth image. Figure 10b,c show, respectively, the reconstructed images by GAN and SAGAN with 10% noise added. The RMSE and SSIM details are presented in Table 2. Observation shows that SAGAN overwhelms GAN in reconstructing the dielectric coefficient distribution of objects, although it has some marginal errors.

**Figure 11:**
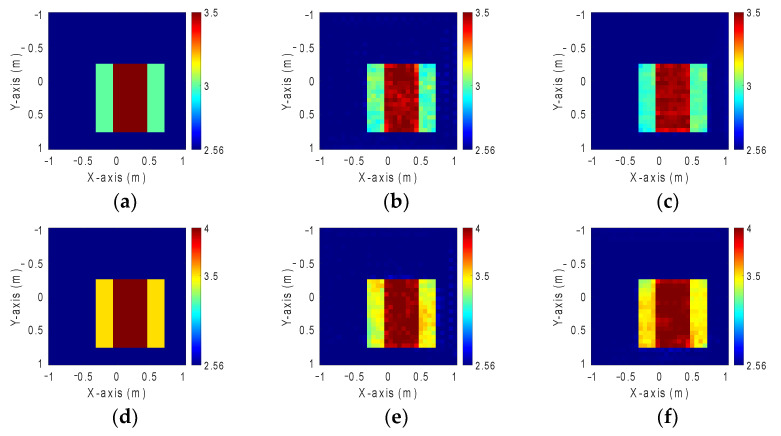
Reconstructed image at 10% noise level for different dielectric coefficient distributions. (**a**) Ground truth with dielectric coefficient distributions between 3 and 3.5. (**b**) Reconstructed image by GAN with dielectric coefficient distributions between 3 and 3.5. (**c**) Reconstructed image by SAGAN with dielectric coefficient distributions between 3 and 3.5. (**d**) Ground truth with dielectric coefficient distributions between 3.5 and 4. (**e**) Reconstructed image by GAN with dielectric coefficient distributions between 3.5 and 4. (**f**) Reconstructed image by SAGAN with dielectric coefficient distributions between 3.5 and 4.

In this part, we have compared different dielectric constant distributions between 3 to 3.5 and 3.5 to 4 with 10% noise. The simulation environment and training parameters are the same as case A. Figure 11a,d show the ground truth image. Figure 11b,c,e,f show the reconstructed image by GAN and SAGAN with 10% noise level, respectively. Relative RMSE and SSIM are listed in Table 3. It is seen that the accuracy and clarity for the large permittivity objects are worse than those for the small permittivity objects.

### 4.3. GAN and SAGAN Performance Comparison for Reconstruction Permittivity between 4 and 4.5 with 10% Noise Level

**Figure 12:**
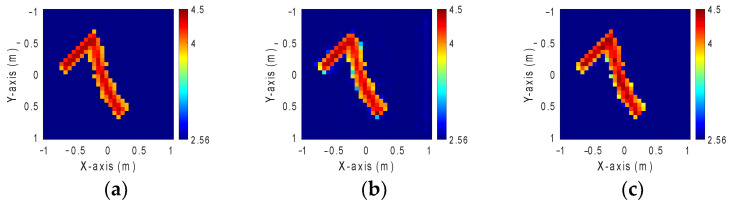
Permittivity from 4 to 4.5. (**a**) Ground truth. (**b**) Reconstructed image by GAN with 10% noise. (**c**) Reconstructed image by SAGAN with 10% noise.

The Modified National Institute of Standards and Technology database (MNIST) includes a substantial collection of handwritten digits from 0 to 9. Each image in the database is sized at 28 × 28 pixels, with 10,000 images available for each digit, resulting in a total of 70,000 images. The dataset is structured such that every 50 consecutive images represent a distinct handwriting style, and each style is rotated at 50 different angles. Due to its simplicity and widespread use in developing various neural network architectures for image processing, MNIST has become a common choice for training such networks. In this case, we distribute the dielectric coefficient between 4 and 4.5 within a simulated environment with 32 transmitters and 32 receivers deployed. A 10% Gaussian noise is added to each transmitter–receiver pair. Handwritten digits (0–9), with 50 images each, are randomly selected from the MNIST database to form a total of 500 images for each scenario. This dataset is also partitioned into three subsets as follows: 90% for training, 5% for validating, and 5% for testing to accelerate the process. The dielectric constant distribution of those handwritten digits estimated prior by the BPS method is fed into GAN and SAGAN models for comparative analysis. Figure 12a illustrates the ground truth image, while Figure 12b,c present the reconstructed images by GAN and SAGAN, respectively, with 10% noise added. The RMSE and SSIM are detailed in Table 4.

### 4.4. GAN and SAGAN Performance Comparison for Reconstruction Permittivity Between 4.5 and 5 by Case C Model

**Figure 13:**
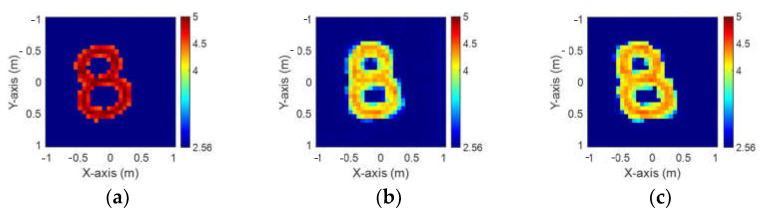
Permittivity from 4.5 to 5. (**a**) Ground truth. (**b**) Reconstructed image by GAN with 10% noise. (**c**) Reconstructed image by SAGAN with 10% noise.

In this scheme, we implement the training model of Case C with 10% noise to divide the dielectric constant between 4.5 and 5 in order to assess the effectiveness of our proposal. Figure 13a is the ground truth image. Figure 13b,c are the images reconstructed by GAN and SAGAN, respectively, with 10% noise added. The corresponding RMSE and SSIM are given in Table 5. Numerical results reveal that GAN is relatively vague compared to SAGAN in reconstructing the contours for the digit eight.

## 5. Conclusions

The 2D inverse scattering problem is being investigated in this paper. We have compared two distinct neural network architectures, GAN and SAGAN, to reconstruct dielectric objects buried in half-space. The object is illuminated with TM-polarized waves in the lower-half plane, and the scattered field is measured from the upper-half side. BPS is used to calculate the initial size and position of the image through the measured scattered field. Ultimately, accurate permittivity can be successfully reconstructed in half-space using both GAN and SAGAN. According to our numerical results, under the same training parameters, the reconstruction results of our proposed SAGAN surpass those of GAN, regardless of the shape types of the object or the distribution of the dielectric constant. It is also realized that despite SAGAN exhibiting superior performance over GAN, it requires a longer training time. In future work, we tend to implementing SAGAN to more difficult simulation environments and reconstruct more complex objects. In addition, we also consider applying SAGAN to the more advanced Switch Transformer architecture expert system.

## Figures and Tables

**Table 1:** RMSE and SSIM of permittivity from 3 to 3.5 with 20% noise added.

	GAN	SAGAN
RMSE	2.3%	1.76%
SSIM	89.6%	94.5%

**Table 2:** RMSE and SSIM of permittivity from 3.5 to 4 with 10% noise added.

	GAN	SAGAN
RMSE	2.96%	2.45%
SSIM	79.8%	95.6%

**Table 3:** RMSE and SSIM with 10% noise level for different dielectric coefficient distributions.

Performance	3–3.5		3.5–4	
	GAN	SAGAN	GAN	SAGAN
RMSE	0.94%	0.89%	2.96%	2.45%
SSIM	97.6%	98.9%	79.8%	95.6%

**Table 4:** RMSE and SSIM of permittivity from 4 to 4.5 with 10% noise added.

	GAN	SAGAN
RMSE	2.48%	1.29%
SSIM	88.1%	98.8%

**Table 5:** RMSE and SSIM of permittivity from 4.5 to 5 with 10% noise added.

	GAN	SAGAN
RMSE	12.75%	11.3%
SSIM	72.9%	78.1%

## Data Availability

Not applicable.
